# Local sustainability and scaling up for user fee exemptions: medical NGOs vis-à-vis health systems

**DOI:** 10.1186/1472-6963-15-S3-S5

**Published:** 2015-11-06

**Authors:** Jean-Pierre Olivier de Sardan, Aïssa Diarra, Félix Yaouaga Koné, Maurice Yaogo, Roger Zerbo

**Affiliations:** 1 Laboratoire d'Etudes et de Recherche sur les Dynamiques Sociales et le Développement Local (LASDEL), BP 12901, Niamey, Niger; 2 École des Hautes Etudes en Sciences Sociales (EHESS), Paris, France; 3 Institut des Sciences Humaines, Bamako, Mali; 4 AFRICsanté et Université catholique d'Afrique de l'Ouest (Unité universitaire de Bobo-Dioulasso), 01BP298, Bobo-Dioulasso, Burkina Faso; 5 Institut des Sciences des Sociétés, Ouagadougou, 03BP7047, Burkina Faso

## Abstract

Free healthcare obviously works when a partner from abroad supplies a health centre or a health district with medicines and funding on a regular basis, provides medical, administrative and managerial training, and gives incentive bonuses and daily subsistence allowances to staff. The experiments by three international NGO in Burkina Faso, Mali and Niger have all been success stories. But withdrawing NGO support means that health centres that have enjoyed a time of plenty under NGO management will return to the fold of health centres run by the state in its present condition and the health system in its present condition, with the everyday consequences of late reimbursements and stock shortages. The local support given by international NGOs has more often than not an effect of triggering an addiction to aid instead of inducing local sustainability without infusion. In the same way, scaling up to the entire country a local pilot experiment conducted under an NGO involves its insertion into a national bureaucratic machine with its multiple levels, all of which are potential bottlenecks. Only experiments carried out under the "ordinary" management of the state are capable of laying bare the problems associated with this process. Without reformers 'on the inside' (within the health system itself and among health workers), no real reform of the health system induced by reformers 'from the outside' can succeed.

The problems relating to the sustainability of public policies in Africa, especially when the policies benefit from development aid, in the area of health among others, are familiar to researchers and policy-makers. However, as far as user fee exemptions are concerned, debates about these problems have extended well beyond the narrow circle of experts and into the public domain in the countries concerned. Throughout our research, we have observed that the sustainability of free healthcare policies is a major concern of all the actors (health workers, users, managers and senior administrative staff), and an issue that has generated widespread scepticism, especially in Mali and Niger [1,2]. There is general unease about the state's ability to reimburse health centres and to provide essential inputs. The scepticism is fuelled by a two-fold negative experience: decades of incoherent public policies at national level, plagued by bad management and uncertain funding, on the one hand; and the endless U-turns by donors, the double binds of frequent contradictions in their funding policies and the short-term nature of the programmes they enact, on the other [3].

The first years of exemption policies, which were beset by late reimbursements and more or less chronic stock shortages, only added to the scepticism. The disquiet appears to be justified: despite their positive impact in terms of health centre attendance, without funding guaranteed over time, efficient management, secure supply channels and motivated staff, free healthcare policies fall foul of a host of adverse effects at every level of the health pyramid.

## A two-tier service of free care: one NGO-funded, the other state-funded

However, at local level, some health centres have been shielded from these negative effects. These are, in the main, health centres that enjoy technical and financial support from external agencies (international institutions and American or European NGOs).

Free healthcare obviously works when a partner from abroad supplies a health centre or a health district with medicines and funding on a regular basis, provides medical, administrative and managerial training, and gives incentive bonuses and daily subsistence allowances to staff [4-10]. The experiments by Terre des Hommes in Burkina Faso, Médecins Sans Frontières-Belgium in Mali and Médecins du Monde in Niger have all been success stories, a fact that is well-publicized in their promotional literature [11-13]. They prove not only that free healthcare leads to a higher level of attendance at health centres once the financial barrier has been removed, which is hardly surprising, but also that there is not necessarily a decline in quality, indeed, it may even improve, if steps are taken at the same time to provide additional funding, support and quality control, and to motivate staff.

However, not far from these pockets of operational efficiency, the other, state-run health centres, which are totally deprived of such infusions of aid, are hit hard by the consequences of late reimbursements and stock shortages. Furthermore, their staff, who are paid less, poorly trained, demotivated and subjected to the state's deplorable management of human resources, show little sense of purpose in the face of a free service, in which they have no faith. In Mali, for example, according to a survey of perceptions conducted among 579 individuals (health workers and users, in particular): "Stakeholders remained sceptical about the State's ability to provide free treatment, which represents a real financial challenge and presupposes a capacity for organization and control which they feel the State does not have" [1]. Moreover, for the majority of respondents: "The idea of free healthcare is associated with a loss of quality, in terms of both relations with staff and the effectiveness of treatment^" ^[1].

Seen in this light, free healthcare policies in Burkina Faso, Mali and Niger have created new inequalities: on one side of the fence, there are privileged health centres, supported by international NGOs, which are able to apply fee exemptions efficiently without compromising quality and have staff who are better paid and better trained; on the other side, there are the majority of health centres, struggling to survive in a destitute context, with no infusions of aid, and in which fee exemptions only aggravate problems, take away some of the formal and informal resources from staff and threaten the supply of inputs. There is a wide gulf between 'NGO-funded' health centres and 'state-funded' ones. The relative prosperity of the former feeds the bitterness of the latter. This is of course the result of a complex historical process mixing the difficulties of state building in former colonies, the extraverted characteristics of development policies [14], and the new public management techniques of neo-liberal ideologies [15]. The end product is a new form of "rentier state" [16], in which aid dependency is a core feature, and where the delivery of public goods by the state is deeply unsatisfactory in the view of most citizens [3].

## The two levels of sustainability

The infusions of NGO aid are, however, normally temporary, and the health centres supported by it are destined to re-enter the mainstream. In other words, the problem of sustainability also arises in their case at local level. *Local sustainability exists alongside national sustainability*. These two processes are often confused, and many NGOs try to do two things at once: the local support an NGO gives to a health centre or district is often presented as a 'pilot experiment', indicating by this designation that it is intended, where possible, to lead to the creation of a permanent public policy through a process of *scaling up*. Most international NGOs operating in the area of health that support a health centre have no intention of setting up in a particular locality for any length of time, thus they make it clear that their mission is a temporary one and that their objective is to withdraw once they have created the conditions for the continuation of the activities they have introduced there. But is it simply a matter of continuing with the experiment on the same scale, in the same locality (a severing of links at local level), or of extending it to the health system as a whole (scaling it up to the national level)? Whether it should be the country's health authorities who benefit from the results achieved at local level or whether ownership by the district management teams is a viable option is never properly discussed at any length by the international NGOs and the ministry of health, either before the former start their operations or after they wind them up.

In the case of free healthcare, a distinction can be made, both operationally and analytically, between local and national sustainability. This will be our approach here. The three case studies that have been conducted by our research team all bear witness to both the aims and difficulties associated with these two forms of sustainability.

## The three cases

One case has been chosen in each of the three countries where our team has investigated the removal of user fees. The three NGOs were pro-gratuity organizations, locally appreciated and well-known, working at the district level in coordination with the local and national health authorities. Production of data has followed classical fieldwork techniques: immersion in the local setting, knowledge of local languages, case studies, in-depth observations, open interviews with different strategic groups (frontline health personnel, NGO staff, district officers, patients, management committees, community agents, chiefs, mayors, etc.). While most NGOs and international agencies are interested primarily in quantitative inquiries about outputs and outcomes, the comparative advantages of qualitative methods, "opening the black box" [17,18] of the implementation process, are described in other papers of this issue (Olivier de Sardan; Ridde & Olivier de Sardan).

We present here a transversal analysis, emanating from the detailed case studies done by Diarra [19], Koné [20], Yaogo & Zerbo [21]. Methodological details are provided in these studies.

In Burkina Faso, the NGO Terre des Hommes (TDH) became involved in the health district of Tougan from 2008 onwards; using funding from the European Union (ECHO) it provided support in the area of mother and child health and infant malnutrition [22]. It introduced user fee exemptions for pregnant women and children under five and the free management of malnutrition among the under-fives, and appointed community actors to educate people in health matters and to distribute inputs. It also bore the entire sum charged to the women for normal and dystocic deliveries (that is to say the balance of 20% chargeable to the patient after deduction of the 80% state subsidy) on the basis of a third-party payment system (reimbursement being made to health centres) [21]. TDH still has an active role today, however it is no longer involved in the current management of fee exemptions but is only concerned with the job of monitoring, including quality control.

In the district of Kangaba in Mali, as part of a six-year programme, Médecins Sans Frontières (MSF)-Belgium pre-empted official policy in granting free anti-malarial drugs (ACTs) and rapid diagnostic tests (RDTs) for children under five and pregnant women (not only that, but the NGO vigorously campaigned in favour of this policy). It then widened the scope of official exemptions by abolishing all consultation and treatment charges for children, irrespective of the nature of the illness. It introduced a modest flat rate of XOF 200 (USD $0.4) for all other users. In villages that were more than five kilometres from a community health centre (CSCOM), it trained community agents, referred to as 'malaria agents', in the use of RDTs and the free distribution of ACTs to those who tested positive. It also trained the members of health committees (ASACO) so that they could conduct awareness-raising sessions with local populations [20].

In Niger, it was the NGO Médecins du Monde (MDM)-France that provided input in the district of Keita from 2006 to 2011. It introduced, and retained until March 2007, user fee exemptions for pregnant women and children under five - a decision that had been taken by the state but was still to be implemented at a national level - and subsequently supported this policy by compensating for the state's shortcomings, especially in terms of reimbursements, and by providing technical support. From April 2009, the NGO conducted a unique experiment in Niger by exempting childbirth from payment using the system of third-party payment, but also with a pre-funding arrangement and the supply of medical kits [20].

## Local support and small-scale sustainability

In all three cases, the NGOs involved had clearly adopted a strategy aimed at establishing a permanent local structure. In particular, they took great care to collaborate closely with the key operational body at this level, i.e. the district supervisory team. In this case, therefore, there was no question of NGOs working 'in a vacuum', an approach that is only too familiar in traditional humanitarian medical aid and is regularly encountered in various areas of development, including health. What one sees here is the very opposite: i.e. a strategy that provides 'support' and involves ongoing cooperation with the instances that make up the institutional fabric of health: namely, management committees and other community organizations, local health centres, line managers, and the Ministry of Health (this is also true of the support provided by the NGO Help in Dori [23]). While each NGO has of course its own identity and organisational culture (Cf. for instance for MSF [24]), there is a striking convergence of their strategies in the three case studies, concerning their relations with the health system and their support to user fees removal.

This support is therefore intended to be temporary and to prepare for the time when it is no longer available, in other words, when responsibility for the routine management of the health system is handed back. But there's the rub: for, looking at it from another angle, the health system as it currently exists at present in the three countries in question is incapable of really taking over at the local level (and even less so nationally). To be more precise, withdrawing NGO support means that health centres that have enjoyed an all too brief time of plenty under NGO management will return to the fold of health centres run by the state. In real terms, this will entail a decline in the quality of healthcare, for both financial reasons (the loss of the extra money provided by the NGO) and human reasons (the loss of training that came with it and the demotivation of staff).

The problem is a relatively simple one: Given the present state of the health systems in Burkina Faso, Mali and Niger, how can state-run health centres, without any resources or training other than those provided by *the state in its present condition *and *the health system in its present condition*, carry out the same activities as those previously made possible by the involvement of the international NGOs with their human and material resources?

In the case of TDH in Tougan, the system of performance-based-financing (PBF) has been one of the important elements in the NGO's involvement locally and remains so to this day. The system entails a substantial financial contribution (in other words, an on-going subsidy from the NGO) and close supervision to ensure that the mechanisms for distributing and overseeing these bonuses run smoothly (management, book-keeping, quality indices, quarterly appraisals and retrocession fees to management boards - COGES). How could the Burkina health system on its own possibly take over at the national level? A financial projection shows that extending these bonuses to the whole country (just for the under-fives) would add between XOF 9 and 12 billion to the state's annual budget [25]. Admittedly, the current policy of the World Bank is aimed at generalizing this kind of incentive scheme in Africa by allocating significant funding to it (currently they "experiment" with PBF at the level of 12 districts). However, neither the State nor even the World Bank will provide the training and supervision at national level that the TDH provided at district level, and one might well be puzzled by a policy that takes no account of the real state of the health system or of the 'practical norms' of its workers [26,27]. One can only applaud this warning: "*In-depth country-level research may be considered an essential precondition for the introduction of incentive schemes*," [28].

In Mali, the NGO MSF-Belgium in Kangaba provided finance for a number of types of expenditure: the wages of the employees of the community health centre (CSCOM); incentive bonuses for a range of other staff; running costs; the Community Health Association's (ASACO) share of funding for evacuation and referral cases; the wages of 'malaria agents'. It paid XOF 1,000 to the CSCOM for every consultation given and XOF 10,000 to the Referral Health Centre (CSREF) for every child hospitalized with a fever. In addition to its expatriate staff, an administrator and a manager were appointed. It carried out frequent and thorough supervisions and inspections. None of this can be reproduced by Mali's state health system, at least not as things stand at present.

When MSF-B withdrew, three strategies were implemented for the continuation of its work at the local level, however they have all failed:

- The provision of the Community Health Centre (CSCOM) with sufficient working capital for three months in the form of cash and medicines. However, this reserve soon ran out and could in no way secure the long-term viability of the services provided.

- The extraction of a commitment from the municipalities and the *cercle *(second level administrative unit) to devote ten percent of their budget to health and fund evacuation and referral entirely. This commitment, which was negotiated by the MSF-B, has not been honoured by the local politicians.

- The quest for other partners, in other words, for a new source of funding, to take over from the MSF-B. This attempt has not been successful.

In fact, faced with the steady collapse of the facilities it had put in place (and a decline in the number of visits), the MSF-B took the decision to extend its infusions of aid, however through a new structure:

- The creation of a local association, the Medical Alliance against Malaria (AMCP), which brought together Malian health workers previously employed by MSF-B and received European aid via MSF-B; what is involved here, therefore, is the first-level 'Malianization' of foreign involvement with a national NGO now acting as a local sub-contractor for an international NGO that finds the funding (in fact, because of security problems in the Sahel, which have resulted in the departure of many European expatriates, international NGOs have embarked on a similar course of 'nationalizing' their activities by turning to local managers to either run their offices on their behalf or act as subcontractors; but the policy is still designed, funded and steered by the international NGOs).

- The refocusing of activities onto the fight against child malnutrition accompanied by a reduction in the scope of free healthcare along national policy lines (with only anti-malarial drugs and RDTs remaining free of charge).

This case spells out the difficulties of the local long-term continuation of the support provided by an NGO. Far from simply handing over responsibility to the district and, hence, to the Malian health system as planned, the NGO has been forced to continue with its support, but in a different format involving the scaling down its ambitions. This is part of a wider phenomenon encountered right across the development aid spectrum: the support delivered by international NGOs is increasingly outsourced to local NGOs.

To the extent that it enables national managers to be trained and employed, this recent trend is to be welcomed. However, the system only exists because the national NGO is supported by its international counterpart. The local health centres that benefit from this aid continue to enjoy a privileged status (that of being run by an NGO) compared to health centres run by the state. However, dependence on outside help continues. This does not, therefore, solve the problem of long-term sustainability at local level, in other words weaning districts and health centres off outside support and re-incorporating them into the 'normal' state system.

The question as to whether the support given by international NGOs to districts and local health centres in order to widen access to free healthcare gradually leads to weaning, or whether it has the completely opposite effect of triggering an addiction to aid is one that is worth raising. The same question applies, moreover, above and beyond free healthcare - and even health itself - to all forms of local development, i.e. direct support that is provided by outside partners to local organizations and institutions via a 'micro-project' type programme or via 'civil society organizations' acting as sub-contractors.

The extension of the support in a new format was also the option in Niger, however this time it was set up differently and was more the result of chance than a decision by the NGO involved or the district it supported. It was the UNFPA that took over from MDM, not as a conscious choice but because its policy of promoting free deliveries in Niger prompted it to choose a health region that included the district of Keita where MDM had developed its pilot experiment of free deliveries. In this particular case, a change of scale (the UNFPA supported free deliveries in four different regions) and of institution was involved: the assistance was now provided by an international organization from within the United Nations system and not by an NGO.

Nonetheless, this case enables the comparison of two very different types of intervention that could be described as 'localized support' and 'mass assistance'.

1. NGOs such as TDH, MSF-B and MDM offer localized support over a limited area (at district level) with expatriate staff already on the ground and national staff recruited for the purpose. Localized support of this nature makes for greater flexibility, better collaboration with health workers and districts and greater responsiveness; it can also result in local norms being taken into account (for example, the remuneration of birth attendants has been tolerated in Keita despite the fact that the NGO promotes free deliveries). However, it has its limitations: there are times when the decisions of the assisting NGO are at odds with local opinion. In Kangaba, for example, workers and local politicians were very resistant to the extension of free healthcare brought in by MSF; however, their opposition was not expressed openly in case it discouraged outside support, from which both these groups benefited: this is another illustration of the contrast between what is said in public (governed by the strategy of "saying the right thing") and what is said in private (information gathered through qualitative surveys): see in this issue Olivier de Sardan. In a similar fashion, the views of local health workers have not always been considered in Keita where reimbursements by MdM for normal deliveries were considered inadequate. Workers in Tougan felt they were being underpaid by TDH.

2. Intervention by the UNFPA, on the other hand, is on a large scale, and largely confined to the funding of free deliveries and the institutional procedures for achieving this objective. 'Mass assistance' is bureaucratic in nature, and does not offer the close supervision ensured by the local support that NGOs provide. In fact, the same problems often arise through UNFPA funding as through funding by the state: delays in the reimbursement of costs and the inadequacy and unsuitability of medical packs have been recorded since the UNFPA took over from MDM.

## Scaling up

With the UNFPA, the second dimension of long-term sustainability emerges on a real-life scale, i.e. that of scaling up, in the sense of generalizing the local experiment to the entire country. Whereas local sustainability is all about breaking dependency (or "discontinuing the infusion"), the *generalization of a pilot experiment *necessarily involves its insertion into a bureaucratic machine of substantial proportions and, therefore, the designing and implementation of a national mechanism that brings the entire health system into play with its multiple levels, all of which are potential bottlenecks: e.g. funding from above, reimbursement procedures, procurement of inputs, logistics, health workers commitment, interaction with users, informing the public, inspection, supervision and monitoring, etc.

The transition from a local experiment receiving local support to a national policy on a massive scale therefore involves much more than political determination or the existence of an adequate budget. These two necessary conditions are often stressed in the literature on public policy-making [29-34]. However, they are not always found together: indeed, far from it, as free healthcare policies have shown. However, there is also a third condition, which is no more likely to feature than the other two: a health system that is sufficiently effective in its operation to be able to implement this *scaling up *in a satisfactory manner, without any major adverse effects. *The day-to-day functioning of the health system (and of the national health bureaucracy) is, therefore, a strategic variable*. In their present form, the health systems of Burkina Faso, Mali and Niger are totally incapable of providing the same kind of training and supervision to health centres on a national scale as NGOs do at a local level. Moreover, they are often to blame for numerous blockages, again as demonstrated by free healthcare. It is part of a more general issue: "Much of the evidence about what works, however, comes from projects undertaken on a small scale in controlled environments where funding and technical assistance have been sufficient," [35].

What all this means is that there are many aspects to "scaling up". In this issue, Valéry Ridde looks at one aspect that concerns "policy-makers", in particular: before promoting a new public policy, do the authorities or international organizations take account or otherwise of the 'pilot experiments' (usually initiated by NGOs) already undertaken in the matter, and, particularly, of the lessons that can be learnt through the research carried out on the subject? The answer is 'no', and it becomes part of the debate on the very possibility of "evidence-based policy making." In this respect, Sanderson [36] tries to steer an interesting middle way between a naïve positivism that imagines public policy-making to be enlightened by science and the post-modernist rejection of the idea that reason plays any part in determining public policies.

Other aspects are regularly broached by studies dealing with these problems, which, in reality, comprise two sub-sets or two points of entry: the literature on sustainability [37-40] and the literature that deals with "scaling up" [41-43]. Both kinds are essentially normative. They seek to provide a complete list of the variables that might enable (or, conversely, compromise) a policy of sustainability/scaling up, and they try adding them together or arranging them in a variety of frameworks. The health systems of the high and middle income countries are, for the most part, their referents, and their building blocks have been transposed to countries in the low income countries. The many problems (various facets of which are described in this issue) posed by the malfunctioning of national health bureaucracies, the incoherent management of human resources within the health system and the inappropriate behaviour of health workers certainly get a mention from time to time, but this tends to be oblique and expressed euphemistically, without too much emphasis being placed on these issues. For example, Simmons & Shiffman [44] mention the need for a "major change in bureaucratic culture", but say nothing about the empirical content of this bureaucratic culture. While the knowledge gap concerning the daily functioning of African bureaucratic cultures have been tackled recently [45,46], it has not been so much the case in the health domain (the analysis of organisational issues being nevertheless a first step forward [47,48]). This question constitutes a major challenge for any scaling up of a local experiment and for any public policies relating to health in general.

From this point of view, the fact that the pilot experiments on free healthcare have been conducted under an NGO makes them irrelevant to the scaling up process. Only experiments carried out under the "ordinary" management of the state are capable of laying bare the problems associated with this process. Of course it does not mean that an experiment undertaken by the state (and in fact there are very few) will automatically be considered, evaluated and scaled up, far from that. A general observation made by Simmons & Shiffman applies rather well to NGO experiments: "When pilot or experimental projects are tested in social and managerial environments that differ greatly from the setting into which they are to be transferred, a second research phase may be called for in which the innovations are validated in more typical programmatic contexts" [49].

As we have stressed, a local NGO providing assistance locally does not only give financial support, it also gives different kinds of human support which come from outside: e.g. behavioural models, technical supervision, training, supervision and monitoring, leadership, staff management, management of flows and inputs, accounting, etc.

In the context of a potential policy of scaling up these experiments, it is certainly possible with a good dose of optimism to envisage national or international funding being found, in other words, the financial support enjoyed by an NGO-assisted district for implementing a local policy of fee exemption being generalized to the national level. African states such as Burkina Faso, Mali and Niger would perhaps have the means to fund fee exemptions from their budgets if a real political will existed and if other budget choices were exercised. But on the other hand, in the current circumstances, it is completely impossible for the human support provided externally by the NGO to be generalized as well. Indeed, with all their inconsistencies, the constraints that bind them, their practical norms, limitations, shortcomings and contradictions, it is the *current human resources *in the health system that will have to scale up a new policy.

It is precisely the management of human resources (in its widest sense, a sense which goes beyond plain "management") that constitutes one of the main bottlenecks in health systems in Africa, and this is one of the main reasons why they are so dysfunctional. If sustainability is defined as the "organizational routinization" of the behaviour learnt or modified during the pilot experiment [38,49], this dreamt-of or imagined routinization comes up against the all-too-real current routines and red tape that characterize the health system, especially the practical norms that govern the behaviour of the actors. In circumstances like these, it is no longer a matter of hoping for or stimulating the political will of those at the top to implement exemption policies, but of undertaking a complete overhaul of the health system and health bureaucracy.

## Conclusion

There is an obvious difference between projects on paper, designed in American or European institutions by international experts - who are, without doubt, competent in their fields and whose goodwill can hardly be called into question - and projects on the ground in Africa, which come up against the stubborn fact that local and national contexts are nothing like the standard model that has been used as the basis for these projects. This is what has been called the "capability trap": "This capability trap emerges under specific conditions which yield interventions that (a) aim to reproduce particular external conditions considered 'best practice' in dominant agendas, (b) through pre-determined linear processes, (c) that inform tight monitoring of inputs and compliance to 'the plan', and (d) are driven from the top down, assuming that implementation largely happens by edict" [50].

In the case of the NGOs that have supported locally free healthcare and which, during the time of their involvement, have produced results in the locality that are often spectacular and always highly valued by the local people, the ultimate goal of sustainability appears well beyond reach, and for one basic reason, over which these NGOs have no control: the present state of the national health systems of the three countries in question with their financial and human shortcomings. A local sustainability strategy, and even more so a national one, that takes no account of the real state of health bureaucracies and, at the same time, does not attempt to reform them, has little chance of success. This is yet another argument for conducting pilot experiments in health centres run by the state rather than an NGO. However, this hope obviously implies that those within the present health system show a willingness to innovate and experiment at different levels leading to outcomes that can be put into practice. *Hence there is a need to find and support local reformers*. Without reformers 'on the inside' [51-53] (within the health system itself and among health workers), no real reform of the health system can succeed. It is not even inconceivable that an unexpected but positive effect of an NGO presence would be to encourage or support reformers on the inside.

However, this is not at the heart of official NGO agendas. Instead of starting from a documented analysis of the real capacities of the health systems in Burkina Faso, Mali and Niger as they exist at present (an analysis consisting of a diagnosis of their day-to-day functioning, the practical norms of health workers and their likely capacity for adapting to change in a credible and realistic way) or from attempts to find local innovators and reformers, NGO support projects take their own objectives and means of achieving them as their starting point. They then apply these to the *perceived *needs of the local population (in the present case, its health needs) with a view to initiating a local action that is supposed to set an example. This is an illustration of the famous "garbage can" model [54], on which Naudet has drawn for his analysis of the world of development [55]: the trick is to look for problems to fit the solutions which are already available and which one is keen to promote. The outcome appears to be more or less the same in every case. The action is a success locally (within the short timescale of intervention) but its sustainability at local level, and even more so at national level, is a failure (in terms of the much longer timescale of the health system).

As the case studies in Burkina Faso, Mali and Niger have shown, any innovation introduced into the health system should entail a realistic and unadorned understanding of the day-to-day operation of the current system at the locations earmarked for the innovation in question.

Or, to put it another way: any scaling up as well as any new health policy that does not *at the same time *include a reform of the health bureaucracies and the practices reaching down into what happens routinely appears be doomed to sustainability failure.

## List of abbreviations

AMCP: Alliance Médicale Contre le Paludisme (Medical Alliance against Malaria)

ACT : Artemisinin-based Combination Therapy

ASACO : Association de Santé Communautaire (Community Health Association)

CSREF : Centre de Santé de Référence (Referral Health Centre)

COGES : Comites de Gestion (Management Comittee)

CSCOM :Centre de Santé Communautaire (Community Health Centre)

ECHO : European Commission Humanitarian Organisation

MDM : Médecins du Monde

MSF : Médecins Sans Frontières

NGO : Non Gouvernemental Organisation

PBF : Performance-Based Financing

RDT : Rapid Diagnostic Test

TDH Terre des Hommes

UNFPA : United Nations Fund for Population Activities

XOF : CFA Franc

## Competing interests

None

## Authors' contributions

JPOS conceived the idea, wrote the draft and final version of the manuscript. AD, FYK, MY and RZ contributed to conception, study design, data collection and analysis for Niger, Mali and Burkina field sites. All authors revised the paper and approved the final manuscript for submission.

## Declarations

This article has been modified from the chapters **Médecins sans frontières et l'Alliance médicale contre le paludisme au Mali** by F. Koné [56], **Appui de proximité et appui bureaucratique. Autour de deux projets pilotes d'accouchement gratuit au Niger** by A. Diarra [57], **Les exemptions de paiement par l'ONG Terre des hommes dans le district sanitaire de Tougan, Burkina Faso** by R. Zerbo and M. Yaogo [58], and **Addiction à l'aide, sevrage ou impuissance? Les ONG médicales face aux systèmes de santé** by J.-P. Olivier de Sardan [59] in the book *"Une politique publique de santé et ses contradictions. La gratuité des soins au Burkina Faso, au Mali et au Niger"*, J.-P. Olivier de Sardan and V. Ridde (eds), 2014, Karthala, Paris, with the permission of the publisher. The publication of this supplement was funded by a grant from International Development Research Centre (IDRC), Ottawa, Canada.

This article has been published as part of *BMC Health Services *Research Volume 15 Supplement 3, 2015: User Fee Exemption Policies. The full contents of the supplement are available online at http://www.biomedcentral.com/bmchealthservres/supplements/15/S3.
